# Dyspnea and outcome expectations are associated with physical activity in persons with pneumoconiosis: a cross-sectional study

**DOI:** 10.1186/s12890-022-02128-2

**Published:** 2022-09-02

**Authors:** Tomohiro Kawaji, Takashi Hasegawa, Yasushi Uchiyama

**Affiliations:** 1 Department of Rehabilitation, Asahi Rosai Hospital, 61 Hirakocho Kita, Owariasshi, Aichi Japan; 2 Division of Creative Physical Therapy, Field of Prevention and Rehabilitation Sciences, Graduate School of Medicine, Nagoya University, 1-1-20 Daiko-minami, Higashi-ku, Nagoya, Aichi Japan

**Keywords:** Physical activity, Pneumoconiosis, Outcome expectations, Dyspnea, Chronic respiratory disease, Pulmonary rehabilitation

## Abstract

**Background:**

There are various reports on factors associated with physical activity in patients with chronic respiratory diseases. However, there are no studies on the relationship between physical activity and psychological or environmental factors. In this study, we investigated the relationship between physical activity and psychological and environmental factors using questionnaires for patients with pneumoconiosis.

**Methods:**

This cross-sectional study included patients with pneumoconiosis who underwent a pneumoconiosis health examination in 2019. A self-administered questionnaire was used to conduct the study. Physical activity was evaluated using the International Physical Activity Questionnaire, and subjective symptoms [dyspnea and quality of life (QOL)], environmental factors (environment around home and life space), psychological factors (depression, stage of change, self-efficacy, decisional balance, and outcome expectations), and others (e.g., experience with pulmonary rehabilitation) were investigated.

**Results:**

The number of respondents in the study was 185 (men: 171, women: 14). Age, dyspnea, stage of change, self-efficacy, outcome expectations, QOL, depression, decisional balance, and life space were significantly correlated with physical activity. In the multivariate analysis, outcome expectations and dyspnea were extracted as independent factors. In the path analysis, outcome expectations and dyspnea had a direct influence on physical activity. Dyspnea directly impacted not only physical activity but also outcome expectations, stage of change, QOL, life space, and depression.

**Conclusions:**

Dyspnea and outcome expectations were associated with physical activity in patients with pneumoconiosis. To improve physical activity in pneumoconiosis patients, it was suggested that it may be necessary to improve dyspnea and promote an understanding of physical activity.

## Background

Pneumoconiosis is a disease characterized by diffuse fibrosis of the lungs and is a typical work-related respiratory disease [1]. In Japan, pneumoconiosis is prevented and managed in accordance with the Pneumoconiosis Law [2]. In compliance with the provisions of the Pneumoconiosis Law, workers with occupational dust exposure, either currently or in the past, shall be subject to health supervision based on classification within a pneumoconiosis supervision class (Class I to IV). The Japanese classification for the supervision of pneumoconiosis classifies workers affected by pneumoconiosis into Class II or higher. Individuals in Class II or higher can still undergo pneumoconiosis health management after retirement [2]. Pneumoconiosis cannot be completely cured [1]; therefore, interventions, such as pulmonary rehabilitation, are performed based on the symptoms.

Chronic respiratory diseases (CRDs), such as chronic obstructive pulmonary disease (COPD) and interstitial pneumonia (IP), have been reported to have systemic comorbidities in addition to respiratory symptoms. These include muscle weakness, exercise intolerance, and depression [3]. Based on these reports, pulmonary rehabilitation has been proven to be effective [3]. It has also been reported that physical activity (PA) decreases in patients with CRD [3, 4]. PA has been reported to be the strongest predictor of life expectancy in these patients compared to other factors such as respiratory function and dyspnea [5, 6]. Therefore, improving the PA is one of the fundamental goals of pulmonary rehabilitation [7]. However, it has not been proven that pulmonary rehabilitation improves PA, and an effective method for improving PA has not yet been established. This is believed to be partly due to the lack of clarity regarding the factors that influence PA in patients with CRD [8].

Factors influencing PA have been studied in healthy older adults individuals and patients with chronic diseases. In healthy older adults, it is believed that physical, social, environmental, and psychological factors interact in a complex, multidimensional manner [9, 10]. It has been reported that in addition to factors similar to those in healthy older adults, there are also disease-specific factors in patients with chronic diseases [11, 12].

Interventions focusing on psychological factors have been reported to be effective in improving PA in healthy older adults and patients with diabetes. One of these methods is the transtheoretical model (TTM). TTM consists of stages of change, self-efficacy (SE), decisional balance (DB), and outcome expectations (OE), and is a method of intervention tailored to each individual’s readiness for behavior change [9]. Moreover, it has been reported that TTM is effective in improving PA in healthy older adults and patients with chronic disease [13–15]. For pulmonary diseases, most studies on factors related to PA in patients with CRD have been conducted in patients with COPD. Many factors have been reported to be associated with COPD, including personal factors such as age, education, and drinking history; physical factors such as body mass index, and systemic inflammation; and respiratory factors including hyperinflation, respiratory function, and dyspnea [16]. However, few studies have examined psychological and environmental factors. As a result, there is no evidence regarding the critical factors impacting PA in patients with CRD. Therefore, this study aimed to clarify the psychological and environmental factors related to PA in patients with pneumoconiosis.

## Methods

### Study design and participants

This was a cross-sectional study. We conducted the research using a mailed questionnaire. The participants were 500 patients with pneumoconiosis scheduled to undergo a pneumoconiosis health examination at the Asahi Rosai Hospital.

Participants were excluded if they had a Mini-Mental State Examination score ≤ 22 or were contraindicated for pulmonary rehabilitation according to Japanese guidelines (heart disease, uncontrolled hypertension, acute systemic disease and fever, severe renal/liver dysfunction/orthopedic disease/mental illness, recent pulmonary infarction/acute pulmonary heart disease, and severe pulmonary hypertension). Participants with COPD, interstitial pneumonia, and asthma were excluded from the study. In this study, an Explanatory Statement and Consent Form were mailed along with the questionnaire. Participants who were read the explanatory letter and were able to consent to the study were asked to sign the consent form and return it with the questionnaires.

The study was approved by the Bioethics Committee of Nagoya University (approval number: 18–515) and the Ethics Committee of Asahi Rosai Hospital (2019.3). The study was conducted in accordance with the Declaration of Helsinki and written informed consent was obtained from all patients.

### Measurements

#### Demographics, personal factors, and subjective symptoms

Participant characteristics, including age, sex, and pneumoconiosis supervision, were recorded. We also investigated the experience of pulmonary rehabilitation, recognition of pulmonary rehabilitation, and smoking history. Pulmonary rehabilitation recognition was evaluated if the participants knew about pulmonary rehabilitation, using a five-point scale. In this study, the questions about recognition of respiratory rehabilitation were developed by us using a Likert-scale [17]. For smoking history, the presence, number of years, and number of cigarettes smoked were investigated, and the Brinkman index (BI) was calculated. We evaluated dyspnea and quality of life in these patients. We used the modified Medical Research Council (mMRC) dyspnea scale to assess the degree of dyspnea in daily life [18]. Quality of life (QOL) was assessed using the Japanese version of the COPD Assessment Test (CAT). The total points were calculated according to the guidelines [19].

#### Physical activity

PA was evaluated using the Japanese version of the International Physical Activity Questionnaire (IPAQ) short form. We calculated the MET-Minutes/week according to the guidelines. In addition, we divided the data into categories (low/moderate/high) based on the guidelines [20].

#### Psychological factors

We examined depression as a psychological factor. Further, based on the theory of TTM, the stages of change, SE, DB, and OE were researched. The stage of change was used to assess the performance of PA and readiness for behavior change. The stage of change for PA was evaluated at five levels (precontemplation, contemplation, preparation, action, and maintenance) [9].

SE was used to assess self-confidence in PA in a variety of situations. SE for PA was evaluated based on six items (negative emotions, making excuses, exercising alone, accessing exercise equipment, interference from others, and weather). Each item was evaluated on a five-point scale, and the total score was calculated [21].

DB was used to investigate the extent to which the participant experienced the benefits and disadvantages of PA. DB for PA was evaluated based on 10 items. Each item was assessed on a five-point scale and the total score was calculated [22].

OE was used to evaluate expectations and an understanding of the benefits of PA. The OE for PA involved nine items. Each item was rated on a five-point scale. The total score was then calculated [23].

#### Environmental factors

We investigated the environment around the home and a range of activities of daily life. The relationship between the environment around the home and PA was evaluated using the Japanese version of the IPAQ-Environment (IPAQ-E) [24]. The total points were calculated according to the guidelines. The range of activities of daily life was assessed using the Life Space Assessment (LSA) tool [25]. The total points were calculated according to the guidelines.

### Statistical analyses

**Fig. 1:**
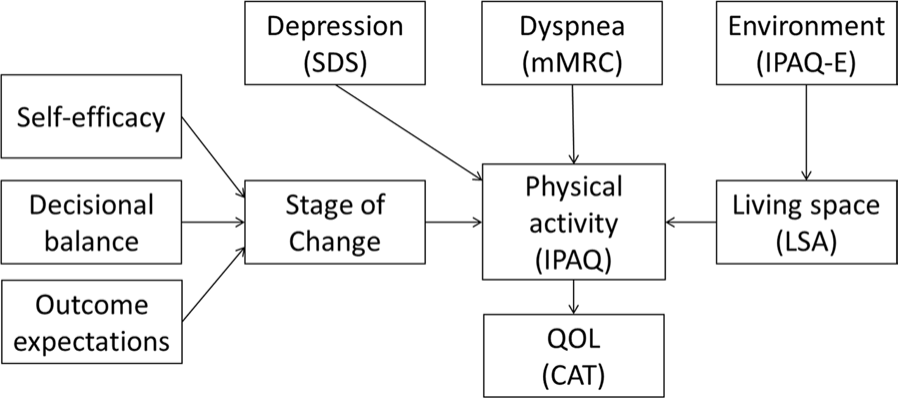
Hypothesized model

The sample size was calculated using an effect size of 0.3, significance level of 0.05, power of 0.80, and number of independent variables of 10. Therefore, the study was conducted with 64 as the target number of participants. Statistical analyses were performed using SPSS software (version 26.0 for Windows; IBM Corporation, Armonk, NY, USA). The assumption of normality was assessed using statistical methods. For all analyses, a *p* value of 0.05 was considered statistically significant. Correlation of PA of patients with pneumoconiosis and each item was analyzed using Spearman’s rank correlation coefficient. Multiple regression analysis (stepwise method) was performed to identify factors that independently affect PA. The data were then path-analyzed using SPSS Amos (Version 26.0 for Windows; IBM Corporation, Armonk, NY, USA). This analysis efficiently and directly allows for the simultaneous modeling and testing of indirect or mediated associations among variables. Figure 1 shows a hypothetical model based on previous research. The hypothetical model was modified based on the results of the correlation and multiple regression analyses, and path analysis was performed to examine suitability.

## Results

Five hundred people who were scheduled to undergo a pneumoconiosis health examination at Asahi Rosai Hospital were considered for this study. Of these, 123 were excluded based on the exclusion criteria and 377 were included. Among the 377 participants, the number of respondents was 185 (men: 171, women: 14), and the response rate was 50.4%. Of the remaining 192 participants, 156 did not return questionnaires and 36 were excluded because of missing values in their questionnaires. The participants’ characteristics are presented in Table 1.

**Table 1:** Characteristics of participants (n = 185)

*Characteristics of participants*	
Sex (men/women)	171/14
Age (years)	78.5 ± 6.1
Pneumoconiosis supervision class (2/3/4)	129/48/8
*Personal factors*	
Pulmonary rehabilitation experience (Yes/No)	18/167
Pulmonary rehabilitation recognition	52/86/11/23/13
(I do not know at all/I do not know much/I cannot say either/I know a little/I know well)	
Smoking history	
(current-smoker/former-smoker/non-smoker)	8/128/62
*Subjective symptoms*	
Dyspnea (mMRC Grade 0/1/2/3/4)	85/53/35/10/2
QOL (CAT 0–40 points)	11 (18–6)
*Physical activities*	
Total score (Mets min/week)	876 (2970–198)
Category classification (low/moderate/high)	86/52/47
*Psychological factors*	
Depression (SDS 20–80 points)	49 (52–44)
Stage of change	49/9/6/12/109
(precontemplation, contemplation, preparation, action, maintenance)	14 (18–10)

Data are shown as mean ± SD for those with normal distribution and as mean median (75–25%) for non-normal distribution

*SD* Standard deviation, *mMRC* Modified Medical Research Council, *CAT* COPD assessment test

Ninety percent (n = 167) had no experience of pulmonary rehabilitation, and 75% (n = 138) were unaware of pulmonary rehabilitation. Regarding subjective symptoms, 54% (n = 100) had dyspnea of mMRC grade 1 or higher. Regarding PA, 47% (n = 86) were in the low activity group. For the stage of change, 65% (n = 121) were at the action and maintenance stage and regularly performed PA.

The results of the correlation analysis between PA (IPAQ total score) and each item are shown in Table 2. PA was significantly negatively correlated with age, dyspnea, QOL, and depression (age, r = − 0.26, *p* = 0.000; dyspnea, r = − 0.34, *p* = 0.000; QOL, r = − 0.33, *p* = 0.000; depression, r = − 0.15, *p* = 0.037). Furthermore, there were significant positive correlations with living space, stage of change, SE, OE, and DB (living space, r = 0.31, *p* = 0.000; stage of change, r = 0.43, *p* = 0.000; SE, r = 0.33, *p* = 0.000; OE, r = 0.36, *p* = 0.000; DE, r = 0.19, *p* = 0.010). The stage of change was moderately correlated, but the other items were weakly correlated.

**Table 2:** Correlation between physical activity and scale scores (n = 185)

Variables	r	*p* value
Age	− 0.26	0.000**
Dyspnea (mMRC)	− 0.34	0.000**
QOL(CAT)	− 0.33	0.000**
IPAQ-E	0.14	0.052
LSA	0.31	0.000**
Depression (SDS)	− 0.15	0.037*
Stage of change	0.43	0.000**
SE	0.33	0.000**
OE	0.36	0.000**
DB	0.19	0.010*

Spearman correlation (r), *p < 0.05; **p < 0.01

*mMRC* Modified Medical Research Council, *CAT* COPD assessment test, *IPAQ-E* International Physical Activity Questionnaire-Environment, *LSA* Life space assessment, *SDS* Self-rating depression scale, *SE* Self-efficacy, *OE* Outcome expectations, DB Decisional balance

The results of the multiple regression analysis are presented in Table 3. Multiple regression analysis was performed using PA (IPAQ total score) as the dependent variable and item significant in the correlation analysis as independent variables (Age, Dyspnea, QOL, LSA, depression, stage of change, SE, OE, DB). OE and dyspnea were the independent factors in the multiple regression analysis. The model was statistically significant (*p* < 0.05) and explained 20% of the variance in PA (adjusted R2 = 0.20). The standardized beta coefficients indicated that both OE (β = 0.25, *p* < 0.05) and dyspnea (β = − 0.15, *p* < 0.05) were associated with PA.

**Table 3:** Results of multiple regression analyses with mean daily physical activity as the dependent variable

	Unstandardized coefficient		Standardized	*p* value	R^2^	R^2^-change
	B	SE	Β			
(Constant)	− 1421.4	1443.5				
OE	141.0	39.8	0.26	0.001		
mMRC	− 537.4	198.9	− 0.20	0.008		
					0.20	

*mMRC* Modified Medical Research Council, *OE* Outcome expectations

**Fig. 2:**
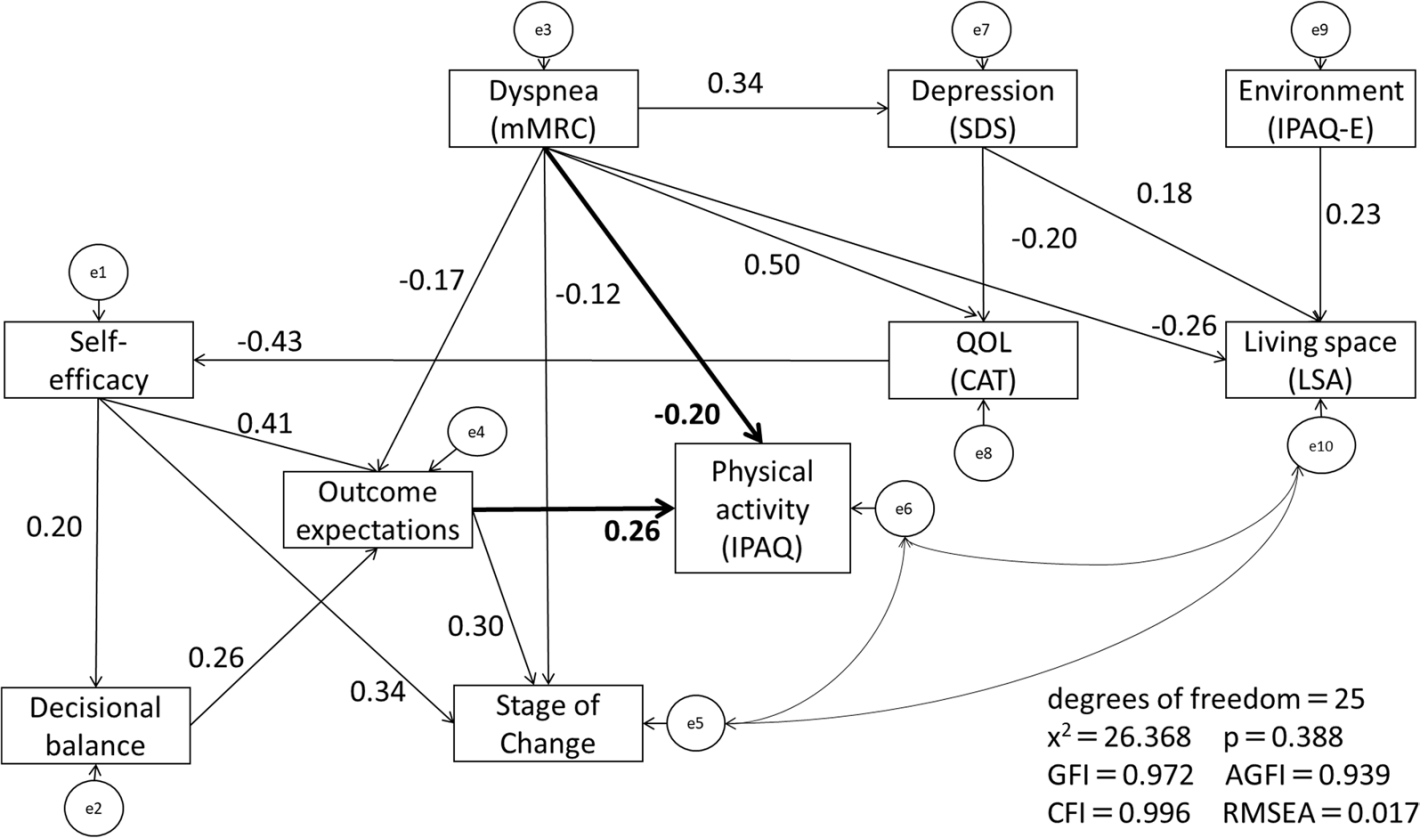
Final model and path coefficients. The strength of this relationship is shown by the path coefficient (standardized regression coefficient)

The path analysis results are shown in Fig. 2. Based on the results of multiple regression analysis in this study, the hypothesized model was modified with dyspnea and OE as covariates of PA (IPAQ total score). And based on the results of the correlation analysis, the hypothesized model was modified with SE, DE, stage of change, depression, quality of life, and living space as mediating variables for PA (IPAQ total score). The path model represented an excellent fit for the data. OE (standardized estimate = 0.26) and dyspnea (standardized estimate = − 0.20) had a direct effect on PA. SE and DB indirectly affected PA through the OE. Furthermore, dyspnea did not only affect PA, but also affected the standardized estimates of OE (− 0.17), stage of change (− 0.12), QOL (0.50), living space (− 0.26), and depression (0.34) directly.

## Discussion

The purpose of this study was to clarify the relationship between PA and psychological and environmental factors in patients with pneumoconiosis. We were able to ascertain the comprehensive relationship between them by conducting a path analysis.

Regarding psychological factors, OE directly affected PA. SE and DB indirectly affected PA, with OE as a mediating factor.

The association between PA and psychological factors in patients with CRD has been reported to be associated with SE [26, 27]. SE represents confidence in performing an exercise [9]. SE has been reported to be associated with PA not only in patients with CRD, but also in a variety of participants, including healthy older adult persons and patients with diabetes. Therefore, it is assumed that behavior change is dependent on SE [9]. In this study, SE was correlated and associated with PA, similar to findings of previous reports. However, the psychological factor that had a direct impact on PA was OE. OE is an understanding of the benefits of PA and the associated expectations. In TTM, it is considered a factor that mediates behavioral change [9]. It has been reported that OE is related to PA in healthy older adults and patients with chronic disease [28–31]. The lack of knowledge about the appropriate PA to perform may explain why OE had more impact on PA than SE. Most of the participants in this study were in the action or maintenance stage of behavior change and reported that they exercised habitually. However, the PA determined by the IPAQ classified them in the low activity group. In other words, there was a difference between subjective exercise and actual PA. Therefore, it is likely that OE, which represents the understanding of PA, was more influential than SE, which represents confidence in performing PA. One possible reason for the lack of knowledge about the required PA is the low implementation rate of pulmonary rehabilitation. The rate of implementation of pulmonary rehabilitation among the participants in this study was lower than that reported for home-based patients with COPD in Japan [32]. Thus, it is assumed that patients with CRD have few opportunities to be educated on the importance, the required amount, and the intensity of PA.

Dyspnea is the most common symptom of CRD [33]. In patients with CRD, dyspnea is reported to be a fundamental symptom that limits PA because patients with dyspnea consciously or subconsciously avoid daily activities [34, 35]. In addition, dyspnea has been reported to affect a variety of symptoms which may be elicited using a question-prompt list such as symptoms of depression. It has been empirically reported that dyspnea causes a vicious cycle of decreased activity and decreased physical function, resulting in further increases in dyspnea [18]. Recently, this vicious cycle has been demonstrated in patients with COPD using path analysis [36]. The results of the present study indicate that dyspnea directly affects PA in patients with pneumoconiosis, as it does in patients with other CRDs. Furthermore, the results of the path analysis showed that dyspnea not only affected PA, but also impacted many other health-related items, such as depression and QOL. Hence, dyspnea was found to be a fundamental factor affecting PA and many other health-related items in patients with pneumoconiosis. Pulmonary rehabilitation has been reported to improve dyspnea in patients with CRD such as COPD and IP [18, 37]. The results of this study also show that pulmonary rehabilitation can improve dyspnea in pneumoconiosis patients, which may also improve QOL and other symptoms.

There was a significant correlation between LSA and PA when environmental factors were evaluated. LSA has been reported to be associated with PA in older adults, and the results were similar to those of previous studies [38, 39]. However, the environment surrounding the home (IPAQ-E) was not associated with PA in this study.

The environment around the home has been reported to be associated with PA and the number of steps in healthy older adult people and patients with various diseases such as knee and hip osteoarthritis (OA) [25, 40–42]. In this study, unlike in previous reports on older adult persons, there was no relationship between PA and the home environment. This may be a unique trend in patients with respiratory disease. This means that patients with respiratory diseases may decrease their PA if they have strong subjective symptoms (dyspnea), regardless of the environment around their home. In patients with OA of the knee or hip, PA has been reported to be associated with the environment surrounding the home, but the influence of physical factors such as pain was stronger [42]. It was believed that subjective symptoms (dyspnea) may have an even stronger influence on PA in patients with respiratory diseases than in patients with other diseases.

We previously reported that older adult patients with pneumoconiosis have greater lower limb muscle strength than healthy older adult patients, even when PA is similar, and that pulmonary rehabilitation is necessary. The results of this study also suggest that pulmonary rehabilitation is necessary for patients with pneumoconiosis. Pulmonary rehabilitation has been reported to improve dyspnea [43]. However, improvement in dyspnea alone is not sufficient to improve PA, and patient education on psychological factors is also important. Until now, patient education in pulmonary rehabilitation has often focused on SE. OE is a prerequisite for behavioral change, and it is believed that both SE and OE need to be improved in order for behavioral change to occur [44]. Therefore, OE is a psychological factor that should be incorporated into intervention programs aimed at promoting PA [44, 45]. Therefore, in addition to conventional pulmonary rehabilitation, patient education is needed to improve OE and patient understanding of PA.

There are several limitations to this study. Because the study design was cross-sectional, it was not possible to estimate causal relationships. In addition, because the study was conducted at a single site, selection bias could have occurred. Therefore, longitudinal studies such as those assessing changes owing to interventions and multicenter studies need to be conducted.

## Conclusion

Dyspnea and OE were associated with PA in patients with pneumoconiosis. This study suggested that it may be necessary to improve dyspnea and promote an understanding of physical activity to improve physical activity in pneumoconiosis patients.

## Data Availability

The data that support the findings of this study are available from the corresponding author, Yasushi Uchiyama, upon reasonable request.

## Abbreviations

CAT: COPD assessment test
COPD: Chronic obstructive pulmonary disease
CRD: Chronic respiratory disease
DB: Decisional balance
IP: Interstitial pneumonia
IPAQ: International Physical Activity Questionnaire
IPAQ-E: International Physical Activity Questionnaire-Environment
LSA: Life space assessment
mMRC: Modified Medical Research Council
OE: Outcome expectations
PA: Physical activity
QOL: Quality of life
SE: Self-efficacy
TTM: Transtheoretical model
